# Supplementary material for: Towards the Elucidation of the Pharmacokinetics of Voriconazole: A Quantitative Characterization of Its Metabolism
Source: Pharmaceutics. 2022 Feb 22;14(3):477. doi: 10.3390/pharmaceutics14030477 (PMC8948939; doi:10.3390/pharmaceutics14030477)
Supplement: Supplementary file 1 [file pharmaceutics-14-00477-s001.zip › pharmaceutics-1494919-supplementary.pdf]

pharmaceutics

Supplementary Materials

Towards the Elucidation of the Pharmacokinetics of Voriconazole: A Quantitative Characterization of Its Metabolism

Josefine Schulz [1], Antonia Thomas [1], Ayatallah Saleh [1,2], Gerd Mikus [1,3], Charlotte Kloft [1,†] and Robin Michelet [1,*,†]

[1] Department of Clinical Pharmacy & Biochemistry, Institute of Pharmacy, Freie Universitaet Berlin, Kelchstraße 31, 12169 Berlin, Germany
[2] Graduate Research Training Program PharMetrX, 12169 Berlin/Potsdam, Germany
[3] Department of Clinical Pharmacology and Pharmacoepidemiology, University Hospital Heidelberg, Im Neuenheimer Feld 410, 69120 Heidelberg, Germany
* Correspondence: robin.michelet@fu-berlin.de; Tel.: +49-30-838-50659
† These authors contributed equally to this work

Supplementary Section S1. Marker reactions for CYP2C19, CYP2C9 and CYP3A4

The recommended marker reactions of the 4-hydroxylation of S-mephenytoin for CYP2C19, 4- hydroxylation of diclofenac for CYP2C9 and 1-hydroxylation of midazolam for CYP3A4 [1,2] were used to determine (i) intersystem extrapolation factors (ISEF), (ii) assess the enzyme specificity of the inhibitors loratadine, sulfaphenazole and ketoconazole and (iii) evaluate VRC and its metabolites as inhibitors. Essential preliminary investigations had confirmed the reaction linearity of metabolite formation and the absence of substrate depletion and were performed in the same laboratory under identical conditions or based on the manufacturer's information.

(i) Experiments for the determination of Michaelis-Menten kinetics in HLM were performed at protein concentrations of 0.02, 0.04 and 0.05 mg/mL for the marker reactions of CYP2C19, CYP2C9 and CYP3A4 (n = 3 each), respectively. Two samples were taken from each incubation after 15 and 25 min for investigations on CYP2C19, after 5 and 10 min for CYP2C9 and after 7 and 12 min for CYP3A4, respectively. For S-mephenytoin 4.58, 9.16, 13.7, 18.3, 36.7, 45.8, 91.6 and 458 µM, for midazolam 0.307, 0.767, 1.53, 3.07, 6.14, 9.21, 20.0 and 30.7 µM and for diclofenac 1.69, 3.38, 6.75, 10.1, 13.5, 27.0, 67.5 and 169 µM were applied as substrate concentrations.

For investigations in rhCYP concentrations of 2.5 pmol/mL rhCYP2C19 (n = 3), 2 pmol/mL rhCYP2C9 (n = 6) and 12.5 and 25 pmol/mL rhCYP3A4 (n = 6) were applied, respectively. Sampling time points were identical to those in HLM as were substrate concentrations of S-mephenytoin and midazolam. For diclofenac a lower substrate concentration of 0.844 µM was included in exchange for the 10.1 µM level.

(ii) The substrates S-mephenytoin, diclofenac and midazolam were applied at concentrations of 45.8, 10.1 and 6.1 µM, respectively, and incubated with HLM at a concentration of 0.2 mg/mL for 15 and 25 min (n = 2). Each probe substrate was incubated in the presence of each inhibitor.

(iii) Incubations for the evaluation of inhibitory effects of VRC, NO and OH-VRC were performed at concentrations of 0.2, 0.08 and 0.05 mg/mL HLM for CYP2C19, CYP2C9 and CYP3A4, respectively. IC_{50} and IC_{50} shift assays were performed at substrate concentrations of 55.0 µM S-mephenytoin, 4.73 µM diclofenac and 4.60 µM midazolam, which was close to the K_M of the reaction. Reaction times were identical to those described under (i).

Table S1. Physiological parameters taken from literature to perform *in vitro in vivo* extrapolation.

Parameter	Value	Reference
CYP enzyme abundance per milligram liver		
CYP2C19	11 pmol/mg	[3]
CYP2C9	61 pmol/mg	
CYP3A4	93 pmol/mg	
Microsomal protein per gram liver (MPPGL)	39.46 mg/g	[4]
Liver mass	1561 g	[5]
Hepatic blood flow (Q_H)	1450 mL/min	[6]
Fraction unbound in plasma (fu_P)	0.5	[7]
Blood to plasma ratio (BP)	1	[8]

Table S2. Specificity of the CYP2C19, CYP2C9 and CYP3A4 inhibitors loratadine, sulfaphenazole and ketoconazole on the marker reactions of the respective enzymes (CYP2C19: 4-hydroxylation of S-mephenytoin, CYP2C9: 4-hydroxylation of diclofenac, CYP3A4: 1-hydroxylation of midazolam) determined as remaining reaction velocity compared to a control incubation without inhibitor in human liver microsomes (n = 3–4). The colors illustrate the interpretation of the specificity: green—high degree of specificity; red—relevant cross-inhibition; orange—ambiguous results; see main text for further explanation.

Enzyme	Marker Reaction	Remaining Reaction Velocity in the Presence of the Inhibitor		
		Loratadine, %	Sulfaphenazole, %	Ketoconazole, %
CYP2C19	S-mephenytoin 4-hydroxylation	<5.0*	77.2	92.2
CYP2C9	Diclofenac 4-hydroxylation	41.7	6.34	84.9
CYP3A4	Midazolam 1-hydroxylation	106	96.9	37.3

* 4-hydroxymephenytoin concentration below lower limit of quantification.

Table S3. Michaelis-Menten kinetic parameters for the marker reactions of CYP2C19, CYP2C9 and CYP3A4 in human liver microsomes (HLM) and recombinant human CYP2C19, CYP2C9 and CYP3A4 (rhCYP).

Enzyme	Reaction	Enzymatic System	K_M (95% CI) (μM)	$V_{max,HLM}$ (95% CI) (pmol/min·mg) or $V_{max,rhCYP}$ (95% CI) (pmol/min·pmol)	$CL_{int, HLM}$ (μL/min·mg) or $CL_{int, rhCYP}$ (μL/min·pmol)
CYP2C19	S-mephenytoin 4-hydroxylation	HLM	53.0 (45.6 – 60.4)	44.7 (42.23 – 47.2)	0.845
		rhCYP2C19	22.1 (17.2 – 27.3)	7.10 (6.59 – 7.62)	0.321
CYP2C9	Diclofenac 4-hydroxylation	HLM	4.15 (3.61 – 4.70)	1693 (1639 – 1746)	408
		rhCYP2C9	2.29 (1.40 – 3.17)	13.5 (12.4 – 14.6)	5.89
CYP3A4	Midazolam 1-hydroxylation	HLM	4.45 (3.56 – 5.35)	559 (523 – 595)	126
		rhCYP3A4	1.96 (1.49 – 2.44)	1.67 (1.56 – 1.78)	0.850

CI—confidence interval; CL_{int}—intrinsic clearance; K_M—Michaelis-Menten constant; V_{max}—maximum reaction velocity.

Table S4. Overview on the type of inhibition caused by voriconazole (VRC), voriconazole *N*-oxide (NO) and hydroxyvoriconazole (OH-VRC) on the CYP isoenzymes specific reactions of S-Mephenytoin 4-hydroxylation (2C19), diclofenac 4-hdyroxylation (2C9) and midazolam 1-hydroxylation (3A4) and the associated inhibitory constants (K_i) as well as the Michaelis-Menten constants (K_M) and maximum reaction velocities (V_{max}).

Enzyme	Inhibitor	Type of Reversible Inhibition	K_i (95% CI) (µM)	K_M (95% CI) (µM)	V_{max} (95% CI) (pmol/min·mg)
CYP2C19	VRC	Competitive	1.90 (1.70–2.12)	37.0 (34.3–40.0)	36.9 (36.1–37.8)
	NO	Non-competitive	58.6 (46.8–75.2)	33.1 (29.8–36.7)	42.4 (40.8–44.0)
	OH-VRC	Competitive	11.6 (9.65–14.0)	29.7 (27.3–32.2)	41.1 (40.1–42.1)
CYP2C9	VRC	Competitive	2.57 (2.16–3.14)	5.80 (5.14–6.52)	2159 (2089–2230)
	NO	Competitive	5.47 (4.32–7.00)	6.32 (5.60–7.13)	2084 (2016–2154)
	OH-VRC	Competitive	2.80 (2.20–3.61)	7.93 (6.81–9.23)	2251 (2158–2346)
CYP3A4	VRC	Non-competitive	2.75 (2.35–3.22)	3.52 (3.05–4.05)	527 (500–554)
	NO	Non-competitive	5.24 (4.68–5.86)	4.05 (3.65–4.48)	505 (486–525)
	OH-VRC	Non-competitive	2.53 (2.24–2.87)	4.16 (3.71–4.67)	446 (427–466)

CI—confidence interval.

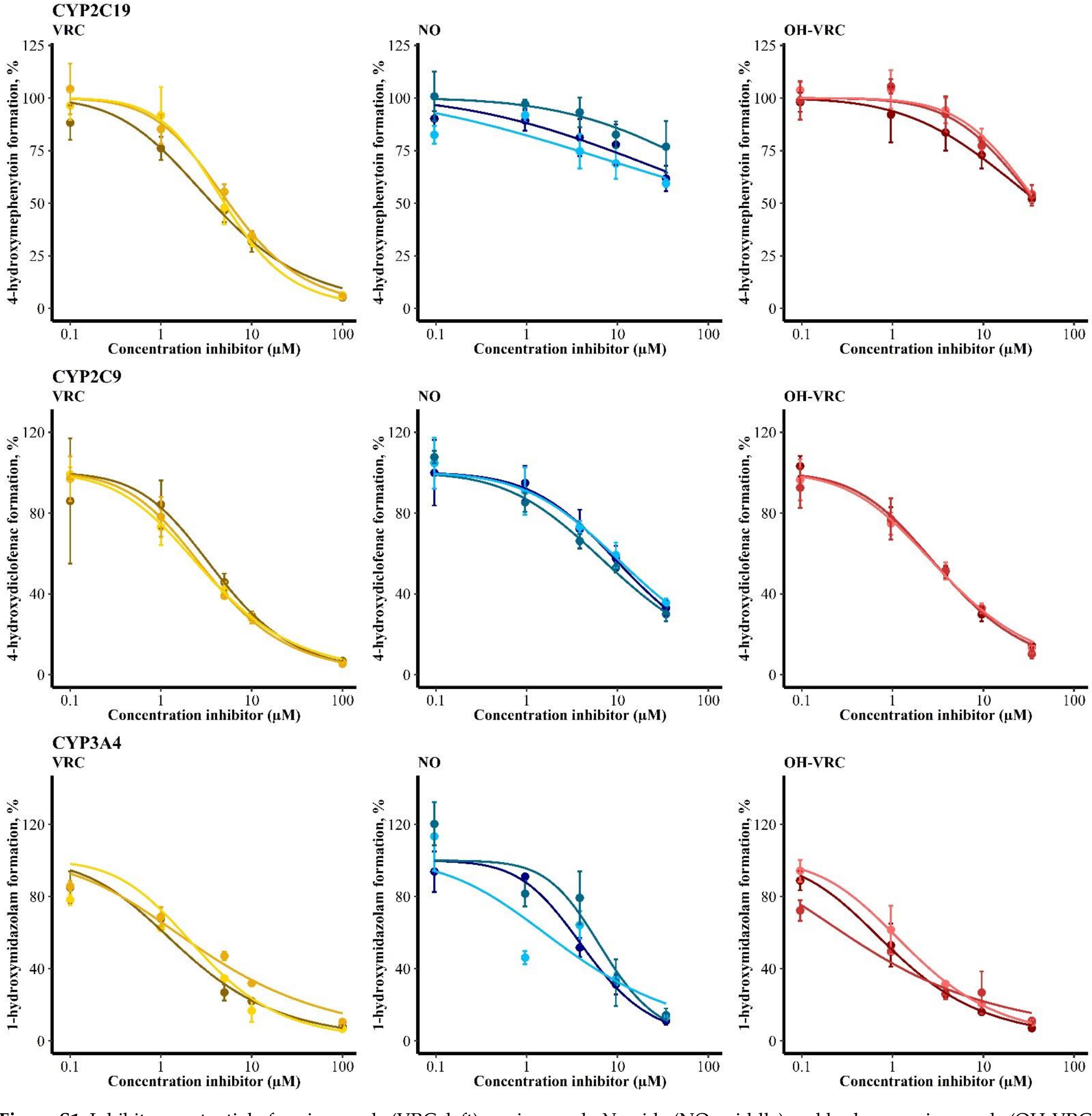

Figure S1. Inhibitory potential of voriconazole (VRC, left), voriconazole *N*-oxide (NO, middle) and hydroxyvoriconazole (OH-VRC, right) on CYP2C19 (top), CYP2C9 (middle) and CYP3A4 (bottom) without a pre-incubation period (dark colors) of human liver microsomes and inhibitor and with a pre-incubation period of 30 min in the absence (medium colors) and presence (light colors) of NADPH re-generating system. Presented is the remaining activity of the respective marker reaction in dependence of the inhibitor concentration.Data points—mean activity (n = 2–4); error bars—standard deviation of activity; solid lines—estimated relation between activity and inhibitor concentration; dark colors—no pre-incubation.

References

1. FDA. Drug Development and Drug Interactions: Table of Substrates, Inhibitors and Inducers. Available online: https://www.fda.gov/drugs/drug-interactions-labeling/drug-development-and-drug-interactions-table-substrates-inhibitors-and-inducers#table1 (accessed on 7 May 2020).
2. European Medicines Agency Guideline on the Investigation of Drug Interactions Available online: http://www.ema.europa.eu/docs/en_GB/document_library/Scientific_guideline/2012/07/WC500129606.pdf (accessed on 18 August 2021).
3. AchourB.; BarberJ.; Rostami-HodjeganA. Expression of Hepatic Drug-Metabolizing Cytochrome P450 Enzymes and Their Intercorrelations: A Meta-Analysis. *Drug Metab. Dispos.* **2014**, *42*, 1349–1356, doi:10.1124/dmd.114.058834.
4. ZhangH.; GaoN.; TianX.; LiuT.; FangY.; ZhouJ.; WenQ.; XuB.; QiB.; GaoJ.; et al. Content and activity of human liver microsomal protein and prediction of individual hepatic clearance in vivo. *Sci. Rep.* **2015**, *5*, 17671, doi:10.1038/srep17671.
5. MolinaD.K.; DiMaioV.J. Normal Organ Weights in Men. *Am. J. Forensic Med. Pathol.* **2012**, *33*, 368–372, doi:10.1097/paf.0b013e31823d29ad.
6. DaviesB.; MorrisT. Physiological Parameters in Laboratory Animals and Humans. *Pharm. Res.* **1993**, *10*, 1093–1095, doi:10.1023/a:1018943613122.
7. VanstraelenK.; WautersJ.; De LoorH.; VercammenI.; AnnaertP.; LagrouK.; SprietI. Protein-Binding Characteristics of Voriconazole Determined by High-Throughput Equilibrium Dialysis. *J. Pharm. Sci.* **2014**, *103*, 2565–2570, doi:10.1002/jps.24064.
8. UtseyK.; GastonguayM.S.; RussellS.; FrelingR.; RiggsM.M.; ElmokademA. Quantification of the Impact of Partition Coefficient Prediction Methods on PBPK Model Output Using a Standardized Tissue Composition. *Drug Metab. Dispos.* **2020**, *48*, 903–916,, doi:10.1124/dmd.120.090498.
